# Understanding performance data: health management information system data accuracy in Southern Nations Nationalities and People’s Region, Ethiopia

**DOI:** 10.1186/s12913-019-3991-7

**Published:** 2019-03-18

**Authors:** Misganu Endriyas, Abraham Alano, Emebet Mekonnen, Sinafikish Ayele, Temesgen Kelaye, Mekonnen Shiferaw, Tebeje Misganaw, Teka Samuel, Tesfahun Hailemariam, Samuel Hailu

**Affiliations:** 1 SNNPR Health Bureau, Hawassa, Ethiopia; 2 Hawassa Health Science College, Hawassa, Ethiopia

**Keywords:** Data quality, Accuracy, SNNPR, Verification factor, HMIS, Performance data, Performance monitoring, Evidence-based decision-making

## Abstract

**Background:**

Health management information system (HMIS) is a system whereby health data are recorded, stored, retrieved and processed to improve decision-making. HMIS data quality should be monitored routinely as production of high quality statistics depends on assessment of data quality and actions taken to improve it. Thus, this study assessed accuracy of the routine HMIS data.

**Methods:**

Facility based cross-sectional study was conducted in Southern Nations Nationalities and People’s region in 2017. Document review was done in 163 facilities of different levels. Statistical Package for the Social Sciences (SPSS) for windows version 20 was used to perform data analysis. Data accuracy was presented in terms of mean and standard deviation of data verification factor.

**Results:**

Though inaccuracy was noted for all data elements, 96.9 and 84.7% of facilities reported institutional maternal death and skilled birth attendance within acceptable range respectively while confirmed malaria (45.4%), antenatal care fourth visit (46.6%), postnatal care (55.2%), fully immunized (55.8%), severe acute malnutrition (54.6%) and total malaria (50.3%) were reported accurately only by about half of facilities. Antenatal care fourth visit was over reported by 24% while total malaria was under reported by 28%. Reasons for variations included technical, behavioral and organizational factors.

**Conclusions:**

Majority of facilities over reported services while under reporting diseases. Data quality should be monitored routinely against data quality parameters quantitatively and/or qualitatively to catch-up country’s information revolution agenda.

## Background

The Health Management Information System (HMIS) is a system whereby health data are recorded, stored, retrieved and processed to improve decision-making [1–4]. HMIS is one of the six core building blocks of the health system and provides data needed for other components (service delivery, health workforce, access to essential medicines, financing, and leadership) [3].

Data delivered through HMIS come from service delivery reports and administrative records kept as part of routine transactions at health facilities and management offices. Data must be collected, processed and transformed, communicated, and used to improve decisions toward improved health outcomes [3, 5].

High quality data are needed to enable safe and reliable healthcare delivery [6] and health facility data are critical inputs to monitor performances [7]. Though different organizations consider different dimensions of data quality, the World Health Organization (WHO) states that the dimensions of data quality are accuracy, validity, reliability, completeness, legibility, timeliness, accessibility, usefulness and confidentiality [5]. But in practice, no health data from any source can be considered perfect. All data are subject to a number of limitations related to data quality such as missing values, bias, measurement error, and human errors in data entry and computation [8] and factors associated with these errors are categorized in to technical, behavioral, and organizational factors [9].

Ethiopia has a three tier health system: primary, secondary and tertiary. Primary health care unit comprises health posts, health centers and primary hospitals. Health centers and health posts are networked by the linkage in which one health center is responsible for supporting approximately five health posts. Secondary level includes general hospitals while tertiary level includes teaching and referral (specialized) hospitals. Ethiopia has been implementing HMIS at all levels of the health system to ensure information use for evidence-based health planning and decision-making [10] with reforms focusing on rationalizing and standardizing the system and information use mechanisms [11].

All levels of health facilities use standard registers and individual cards to record and standard formats to report data. These registers and reporting formats are designed considering services provided at each levels of health facilities and are distributed by federal ministry of health. Except very few hospitals that use computerized data system, all service delivery points use printed materials for recording. Regarding reporting, health posts report to cluster supporting (supervising) health centers (or primary hospitals) which then report to district health office. General and teaching hospitals report to zones where they are located. Some health centers and all hospitals use computer for data entry and analysis. Facilities using computers enter data and submit softcopy while those facilities without computer submit hardcopy to district health office. HMIS reports submitted to district by hardcopy or softcopy are digitalized and shared by higher levels through web system. Except health posts where any of two health extension workers can compile reports, all organizations have person in charge of HMIS activities.

To improve data quality for better decision-making, data quality must be monitored qualitatively and/or quantitatively [5] but there was limited information on the routine HMIS data accuracy in the study area. Therefore, this study assessed the routine HMIS data accuracy in Southern Nations, Nationalities and People’s Region (SNNPR) Ethiopia.

## Methods

Facility based cross-sectional study was conducted in SNNPR in 2017. SNNPR is the third largest administrative region of Ethiopia representing about 20% of the country’s population. From 2007 census, its population was estimated to be 19,170,007 in 2017. It is the most diverse region in the country in terms of language, culture and ethnic background. Administratively, the region is divided into 14 zones, 1 city administration and 4 special woredas. Zones are divided in to woreda and town administrations. Woreda (equivalent to district) is administrative structure in zone with approximate population of 100,000 while special woreda is a woreda that is directly accountable to the region (not included in zone). In 2017, there were 57 hospitals of all type, 736 health centers and 3865 health posts reporting data through routine HMIS.

This study was stand-alone survey, was not linked to community (data verification was done only at facility level), and used both quantitative and qualitative methods. Public health facilities reporting data to government system through the routine HMIS for more than a year were included in the study.

Sample size was determined by using sample size formula for facility survey.

$$ n\kern0.5em =\kern0.5em \frac{{\left[{z}_{\alpha /2}\right]}^2 fq}{{\mathrm{V}}^2\mathrm{p}} $$

Where n = sample size, f = design effect, p = anticipated proportion of facilities with attribute of interest, q = 1-p, V2 = relative variance (square of the relative error) and Z is reliability coefficient at 95% level of confidence.

Assuming p (proportion of health facilities reporting accurate data) to be 50% at 95% level of confidence and considering 20% relative error, design effect of 1.5 and finite population formula, final sample size for all facilities was 138. Distribution of sample size to facility type considered health center to hospital ratio and pairing health center (HC) with health post (HP). For every selected HC, one HP reporting to selected HC was selected.

Sample size was allocated to zones and special woredas proportionally considering existing number of functional facilities. Multi-stage sampling was used to select HCs and HPs while hospitals were selected using simple random sampling. At first level, woredas were selected from zones and at second stage, health facilities were selected from woredas using simple random sampling. For up to three HC-HP pairs, we selected a woreda from zone and for HC-HP pairs more than three, additional woreda was selected. In this way, 25 woredas in addition to determined sample size were included. The overall facilities included were 65 HCs, 65 HPs, 8 hospitals and 25 woreda health offices giving a total of 163 facilities.

At facility level, data accuracy was assessed by comparing source documents and reports while at woreda health office level, accuracy of data entry was assessed by comparing reports from facilities and report sent to higher level through HMIS over the same period.

We considered data of the most recent completed quarter in Ethiopian fiscal year (November 2016, December 2016 and January 2017). To check data accuracy, data elements were selected based on priority and/or weight given by the region for monitoring and evaluation of performance. But administrative reports were not selected because of unavailability of source document to verify. Most administrative reports, for example number of villages free from open defecation, can preferably be verified at community level. Hence, excluded from verification.

Based on these criteria, data elements selected were antenatal care fourth visit (ANC4), skilled birth attendance (SBA), early postnatal care coverage within 7 days (PNC), total malaria (TM), confirmed malaria (CM), tuberculosis case detection (TD), fully immunized for under one year children (FI), institutional maternal death (MD) and new cases of severe acute malnutrition (SAM). Total malaria includes both confirmed and clinically treated malaria cases.

BSc holder nurses and health officers reviewed documents. Training of data collectors (with pre-test) was given for three days. Prior experience on HMIS and data collection was considered in selection of data collectors and supervisors. Daily supervision was also done by principals and supervisors who had master’s degree and above. All collected data were examined for the completeness and consistency of data by principal investigators. Finally, checklists that were incomplete or inconsistent were re-administered. Data was entered, cleaned and analyzed using Statistical Package for the Social Sciences (SPSS) for Windows version 20. Descriptive statistics was used to characterize data quality. Verification factor (VF) was used to describe accuracy of data and expressed in terms of means and standard deviations (SD). VF is fraction of value of data element by re-count to value of data element reported over the same period. Confidence interval (CI) for mean of each data element was calculated using Open Epi version 3.0 at 95%.

A report was considered ‘accurate’ when VF fall between 0.9 and 1.1 (that is ±10% precision). When fraction of re-count to report was less than 0.9 or greater than 1.1, report was considered ‘inaccurate’. When both source and report were zero (0÷0), it was considered 1 (VF = 1) to indicate it was reported accurately but when report was zero for any existing numbers in source document (number÷0), it was considered ‘missing’ as dividing number to zero is undefined. And facilities with VF of missing value were excluded from analysis for specific data element.

For ideal report, re-count and report are equal and VF = 1. The deviation from 1 shows under or over reporting. If report is over, then VF < 1 and if report is under, then VF > 1. The difference between VF of ideal report and observed VF (that is 1-VF) shows under or over reporting.

When there were variations in report and re-count, persons in charge of data were asked for reasons of variations. Reasons for variations were collected qualitatively and summarized manually using descriptive approach.

Ethical clearance was obtained from the regional health bureau Ethical Review Board (Ref. 6-19-2762). Official letter was written to each facility and verbal consent was obtained from each individual respondent and data handler after through explanation of the purpose, benefit, risk and confidentiality of the study. The data was kept anonymous and confidential.

## Results

The study covered a total of 163 facilities: 25 woreda health offices, 65 health centers, 8 hospitals and 65 health posts.

Except institutional maternal death and skilled delivery, other data elements selected were reported inaccurately by majority of facilities assessed. Only about half of facilities reported ANC4 (46.6%), PNC (55.2%), FI (55.8%), TM (50.3%), CM (45.4%) and SAM (54.6%) accurately within 10% precision. Majority of HPs reported selected data elements inaccurately. For instance, ANC4, PNC and FI were reported accurately only by 44.6, 24.6 and 43.1% of HPs respectively. Proportions of facilities (by type) that reported selected data elements accurately were presented in Table 1.

**Table 1:** Proportions of facilities that reported selected data elements accurately, SNNPR, 2017

Data elements	Number and percent of facilities				
	Woreda No (%)	HC No (%)	Hospital No (%)	HP^a^ No (%)	Total No (%)
ANC4	24 (96)	21 (32.3)	2 (25)	29 (44.6)	76 (46.6)
SBA	24 (96)	52 (80.0)	7 (87.5)		83 (84.7)
PNC	24 (96)	46 (70.8)	4 (50.0)	16 (24.6)	90 (55.2)
FI	23 (92)	35 (53.8)	5 (62.5)	28 (43.1)	91 (55.8)
MD	25 (100)	63 (96.9)	7 (87.5)		95 (96.9)
TD	18 (72)	44 (67.7)	6 (75.0)		68 (69.4)
TM	15 (60)	23 (35.4)	2 (25.0)	42 (64.6)	82 (50.3)
CM	15 (60)	16 (24.6)	1 (12.5)	42 (64.6)	74 (45.4)
SAM new cases	7 (28)	44 (67.7)	3 (37.5)	35 (53.8)	89 (54.6)

^a^-HPs do not report SBA, MD and TD

### Verification factor

The mean VF (with SD) of each data element was computed and compared by type of facility. At woreda level, except SAM with 1.14, mean VFs of all data elements were in range of 0.99 to 1.07 which was within 10% precision with small standard deviation (0.00–0.42) while mean VFs of all data elements at health post level, except SAM new cases, were out of 10% precision. The mean VFs of 6 out of 9 data elements at hospital level were also out of 10% precision (Table 2).

**Table 2:** Mean verification factors of data elements by type of facility, SNNPR, 2017

Institutions	Statistics	Data elements								
		ANC4 *n* = 163	SBA *n* = 98	PNC *n* = 162	FI *n* = 160	MD *n* = 98	TD *n* = 92	TM *n* = 161	CM *n* = 158	SAM *n* = 152
Woreda	Mean	0.99	0.98	0.99	1.02	1.00	0.94	1.05	1.07	1.14
	SD	0.04	0.11	0.08	0.08	0.00	0.23	0.39	0.42	0.69
Hospital	Mean	0.73	0.98	0.57	1.02	1.04	1.13	2.30	0.79	1.27
	SD	0.28	0.07	0.49	0.26	0.12	0.40	3.76	0.53	0.47
HC	Mean	0.74	0.96	0.87	0.96	0.97	1.26	1.42	1.04	1.01
	SD	0.30	0.10	0.60	1.23	0.17	1.26	1.68	1.09	0.97
HP^a^	Mean	0.69		0.71	0.77			1.84	0.89	0.95
	SD	0.39		0.58	0.54			4.44	0.53	0.60

^a^-HPs do not report SBA, MD and TD

Descriptive analysis of VF (for all facilities with valid report) showed that except SBA, all data elements had minimum VF of zero and maximum varying for each data element. The difference between VF of ideal or expected report and observed report (1-VF) showed that services like ANC4 had positive value (0.24) indicating 24% over reporting while diseases like TM (− 0.28) and SAM (− 0.02) had negative value indicating 28 and 2% under reporting respectively (Table 3).

**Table 3:** Overall mean VFs of data elements and deviations from ideal value, SNNPR, 2017

Data element	Statistics					
	Min	Max	Mean of VF	SD	(1-VF)	95% CI of 1-VF
ANC4 (*n* = 163)	0.00	1.67	0.76	0.336	0.24^a^	[0.19, 0.29]
SBA (*n* = 98)	0.44	1.16	0.97	0.103	0.03	[0.03–0.05]
PNC (*n* = 161)	0.00	3.06	0.79	0.477	0.21^a^	[0.14, 0.28]
FI (*n* = 159)	0.00	3.33	0.84	0.461	0.16^a^	[0.09, 0.23]
MD (*n* = 98)	0.00	1.33	0.98	0.146	0.02	[0.00, 0.05]
TM (*n* = 159)	0.00	11.53	1.28	1.515	−0.28^a^	[−0.04, − 0.51]
CM (*n* = 158)	0.00	8.33	0.97	0.788	0.03	[−0.09, 0.15]
SAM (*n* = 152)	0.00	8.00	1.02	0.786	−0.02	[− 0.15, 0.10]
TD (*n* = 91)	0.00	2.25	1.02	0.293	−0.02	[−0.08, 0.04]

^a^-mean out of 10% precision

### Reasons for variations

According to HMIS focal persons in charge of data handling, reasons for variations between reports and re-counts were workload, report by phone that were not documented, not registering and/or tallying, poor supervision, illegible data, no or poor feedback, negligence (carelessness), manipulating for competition, poor competency (awareness), not reviewing performance, not sharing experience, not undertaking institutional data quality assessment, lack of commitment, lack of tools (e.g. tally), poor integration, turnover and missing report (losing report). Some of data handlers were not aware of the facts that how errors were made.

## Discussion

This study was designed to assess the routine HMIS data accuracy by comparing report with source document. Though inaccuracy was noted for all data elements, majority of facilities reported institutional maternal death and skilled birth attendant within acceptable range while ANC4, PNC, FI and TM were highly inaccurate. The mean value of VF for majority of data elements varied among different facility types.

Reliable and accurate public health information is essential for monitoring and evaluating health and improving the delivery of health-care services and programs [12–17]. Safe, reliable health and social care depends on access to and use of quality data [6]. Nowadays, technological advancement has increased access to data but the quality of data has been identified as critical area needing intervention [18].

Government of Ethiopia has been implementing HMIS that facilitates data handling and utilization [10] with reforms focusing standardizing tools and process, and information use [11]. This system is usually appreciated as an opportunity to improve data quality and utilization. But, majority of data elements assessed were highly inaccurate. On average, ANC4, PNC and FI were over reported by 24% (95%CI =19–29%), 21% (95%CI =14–28%) and 16% (95%CI =9–23%) respectively while TM was under reported by 28% (95%CI =4–51%). Except SBA, all data elements had minimum verification factor of zero that shows false report or reports from no source which unquestionably affects performance measurement and thereby decision-making. The depth of inaccuracy among facilities was presented in Tables 1, 2, and 3.

In the study area, as part of performance evaluation, government emphasizes on improvements in maternal and child health and reduction of malaria incidence. So, over reporting services and under reporting diseases might indicate attempts to claim better performance. Even though SBA and MD had high evaluation weight, these data elements had low inaccuracy due to intensive data audit during integrated and program specific supportive supervisions. In HMIS, TM consists confirmed and clinical malaria. Higher inaccuracy of TM (VF = 1.28, SD = 1.51) might be due to strict follow-up of clinical malaria (direction that cases should not be treated clinically or cases should be confirmed by malaria tests); that is clinical malaria was not reported (under reported). This indicated that having good system to produce, handle and utilize may not guarantee data quality as reported by the study done in Malawi [19]. As data elements frequently audited during different supervisions had lesser inaccuracy, we suggest using different data elements to verify data quality during supervisions and changing data elements for subsequent auditing.

HMIS implementation is usually challenged by a number of factors that can be categorized in to technical, behavioral, and organizational factors [9]. District-based health information system strengthening implementation assessment in Uganda [20] showed that limited access to computers and internet, inadequate technical support and limited worker force were challenging the system. Assessment of data management and reporting systems in Botswana [21] showed that limited ownership within facilities, lack of training and limited functionality of electronic data management systems were weakness of HMIS. Also in this study, similar challenges of technical, behavioral, and organizational factors were noted. Though system has standardized HMIS, factors related to organizations included workload, poor supervision, no or poor feedback, poor data quality assessment, turnover and lack of tools (e.g. tally). Technical and behavioral factors included reports by phone that were not documented, not registering and/or tallying, illegible data, negligence (carelessness), manipulating for competition, poor competency (skill gap), lack of experience sharing, lack of commitment and missing (losing) report. Furthermore, some of data handlers were not aware of the facts that how errors were made which shows poor technical competency.

Health systems performance cannot be adequately monitored where health information data are incomplete, inaccurate, or untimely [13]. Decisions made using inaccurate data might mislead directions. So, to address these issues, system must design strategies and be watchful to maintain data quality. We believe that if system related factors are well addressed, both technical and behavioral factors at least can be minimized. For instance, strengthening existing supportive supervision can contribute to improvement for organizational, technical and behavioral factors by supporting data quality assessment and assurance practice.

This study was limited in linking facility data with community service utilization as it was stand-alone survey because of limited logistics.

## Conclusions

Majority of facilities over reported services while under reporting diseases. ANC4, PNC, FI and TM were the most inaccurate among data elements assessed. Data quality should be monitored routinely against the data quality parameters quantitatively and qualitatively to catch-up country’s information revolution agenda.

## Abbreviations

ANC4: Antenatal Care (visit) 4
CM: Confirmed Malaria
FI: Fully Immunization
HMIS: Health Management Information System
MD: (Institutional) Maternal Death
PNC: Postnatal Care
SAM: Severe Acute Malnutrition
SBA: Skilled Birth Attendance
SNNPR: Southern Nations Nationalities and People’s Region
SPSS: Statistical Package for the Social Sciences
TD: Tuberculosis Case Detection
TM: Total Malaria
